# Editorial: Artificial Neural Networks as Models of Neural Information Processing

**DOI:** 10.3389/fncom.2017.00114

**Published:** 2017-12-19

**Authors:** Marcel van Gerven, Sander Bohte

**Affiliations:** ^1^Department of Artificial Intelligence, Donders Institute for Brain, Cognition and Behaviour, Radboud University Nijmegen, Nijmegen, Netherlands; ^2^Department of Machine Learning, Centrum Wiskunde and Informatica, Amsterdam, Netherlands

**Keywords:** neural networks, artificial intelligence, computational neuroscience, rate coding, spiking neural networks

## Introduction

In artificial intelligence (AI), new advances make it possible that artificial neural networks (ANNs) learn to solve complex problems in a reasonable amount of time (LeCun et al., 2015). To the computational neuroscientist, ANNs are theoretical vehicles that aid in the understanding of neural information processing (van Gerven). These networks can take the form of the rate-based models that are used in AI or more biologically plausible models that make use of spiking neurons (Brette, 2015). The objective of this special issue is to explore the use of ANNs in the context of computational neuroscience from various perspectives.

## Biological plausibility

Biological plausibility is an important topic in neural networks research. That is, are ANNs simply convenient computational models or do they also inform about the computations that take place in our own brains?

Marblestone et al. carefully lay out the rapid advances in deep learning and contrast these developments with current practice and views in neuroscience. Their main insight is that biological learning may be driven by the optimization of cost functions using successive neural network layers.

A classic question that has haunted ANNs for years is whether backpropagation is biologically plausible (Crick, 1989). Scellier and Bengio introduce Equilibrium Propagation as a new learning framework for energy-based models. The algorithm computes the gradient of an objective function without relying on separate circuits for error propagation that integrate non-local signals.

While acetylcholine (Ach) and dopamine (DA) are neuromodulators that are known to have profound and lasting effects on the neural responses to stimuli, it is unknown what their respective functional roles are. Holca-lamarre et al. develop a neural network model that is combined with the physiological release schedules of ACh and DA.

## Improving performance

Several papers propose new mechanisms to improve the perfomance of ANNs.

Li et al. investigate chunking, which is a phenomenon referring to the grouping of items when performing a memory task, leading to improvements in task performance. The authors show that chunking can have computational benefits as it allows the use of synapses with narrow dynamic range and low precision when performing a memory task.

An important limitation of Hopfield networks is their limited storage capacity. Folli et al. show that by allowing non-zero diagonal elements on the weight matrix, maximal storage capacity is obtained when the number of stored memory patterns exceeds the network size.

McClure and Kriegeskorte introduce representational distance learning (RDL) as a stochastic gradient descent method that drives the representational space of a student model to approximate the representational space of a teacher model.

## Spiking neural networks

An important endeavor in computational neuroscience is to further our understanding of biological and artificial spiking neural networks.

How sensory stimuli relate to the activity of neurons is one of the big open questions in neuroscience, and determining this relationship between the input a neuron receives and the outgoing spike-train has remained a challenge. Zeldenrust et al. propose a new ANN-based method to measure *in vitro* how much information a neuron transfers in this process.

The rate with which spikes are emitted is often mapped to the analog activation values of artificial neurons, but it is well-known that this relationship captures only part of the information processing in real neurons. Carrillo-medina and Latorre develop networks of spiking neurons that operate based on the principles developed for so-called signature neural networks.

How does the central nervous system develop the hierarchy of sensory maps that reflect different internal or external patterns and/or states? Chen shows how simple recurrent and reentrant neuronal networks can discriminate different inputs and generate sensory maps.

### Understanding brain function

ANNs have also been embraced as a new tool for understanding neural information processing in the brain. In this special issue, a number of advances in this area are put forward.

One question is whether supervised or unsupervised neural networks provide better explanations of neural information processing. Testolin et al. taught neural networks to learn an explicit mapping between different spatial reference frames. They show that both network architecture and the employed learning paradigm affect neural coding properties.

An elusive property of our own brains is that we engage in dreaming during sleep. Horikawa and Kamitani used deep neural networks in an effort to decode what people dream about. They found that decoded features from dream fMRI data positively correlated with those associated with the object categories that related to the dream content.

An important question in neuroscience is how neural representations to sensory input are functionally organized. Güçlü and van Gerven show that neural responses to sensory input can be modeled using recurrent neural networks that can be trained end-to-end.

## Conclusion

Neural networks are experiencing a revival that not only transforms AI but also provides new insights about neural computation in biological systems. The contributions in this special issue describe new advances in neural networks that increase their efficacy or plausibility from a biological point of view. A closer interaction between the AI and neuroscience communities is expected to lead to various other theoretical and practical breakthroughs in the years to come.

## Author contributions

All authors listed have made a substantial, direct and intellectual contribution to the work, and approved it for publication.

### Conflict of interest statement

The authors declare that the research was conducted in the absence of any commercial or financial relationships that could be construed as a potential conflict of interest.
